# Amalgam

**Published:** 1879-01

**Authors:** 


### EDITORIAL, ETC.

*Amalgam for Tooth Filling.*—The *Scientific American* in reviewing Drs. Van Denburg's and Truman's articles on "Amalgam" published in the November number of the *Dental Cosmos*, the former favoring the use of amalgam under many conditions, and the latter not being so favorable to its use though not condemning it under certain conditions, remarks as follows:

"So long as the filling of teeth with gold remains a tedious and painful operation, the public generally, since they have to bear the pain, will naturally prefer the employment of plastic fillings, if such fillings can be made to do reasonable service. Can they?

"On this point the professional world is sharply divided, one school of dentists holding that nothing but gold should be used in any case, the other that cheaper and more easily inserted fillings are in many instances, if not always, better than gold. So marked a difference in professional opinion on a point of such vital importance would be a professional disgrace, were it not for the circumstance that the human mouth presents conditions so variable and uncertain that it is impossible to decide in many cases whether the success or failure of an operation is due to the skill of the dentist or his lack of it, to the nature of the filling, to the organic life and condition of the patient's teeth, to the character of the local secretions to which the work is subjected, or to other complications impossible of detection or control. Some salivas seem capable of dissolving anything; other mouths will hospitably receive fillings that could not be trusted at all under the conditions usually prevailing; and even in the same mouth variations in the patient's general health may cause the sudden failure both of filled teeth and of previously sound teeth, that have successfully stood the test of years of usefulness.

"Such being the case it is not surprising that dentists of equal intelligence and skill should be found in opposition.

Probably the question of gold *versus* amalgam will remain in professional chancery until some lucky inventor hits upon a composition approximating dentine and enamel in physical properties, with the ability of pure gold to withstand the action of every possible food or drink or buccal secretion. Meantime it is interesting to watch the shifting aspect of the professional battle—provided one is not called upon to decide upon a filling for his own failing teeth."

The review is to be found in the present number of this JOURNAL.

